# Supplementary material for: Time‑resolved multi-omic analysis of paclitaxel exposure in human iPSC‑derived sensory neurons unveils mechanisms of chemotherapy‑induced peripheral neuropathy
Source: Cell Death Dis. 2026 Feb 10;17(1):211. doi: 10.1038/s41419-026-08445-2 (PMC12921266; doi:10.1038/s41419-026-08445-2)
Supplement: Supplementary file 15 — Dataset 1. [file 41419_2026_8445_MOESM15_ESM.zip › CDD_Open_Data/04_RNA_Seq/Timeline_panel_plots_etc_2023-01-23_1609.zip!/Panel_Plots_etc/Evidence_Plots/post5d/evidence_plot_standard contrasts_msigdb_reactome_5d_PTX_vs_DMSO_ID6_M26917_REACTOME_RAF_INDEPENDENT_MAPK1_3_ACTIVATION.pdf]

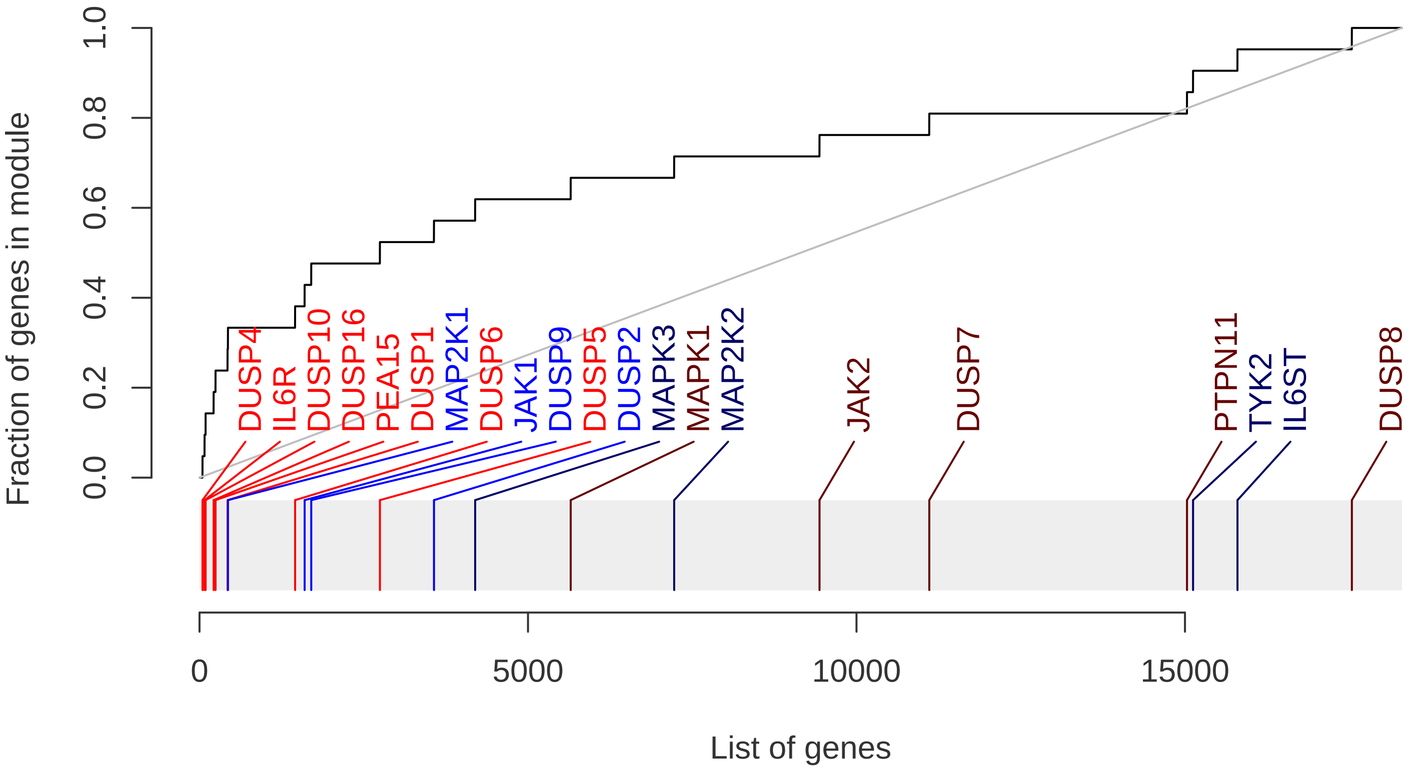

Fraction of genes in module
0.0
0.2
0.4
0.6
0.8
1.0
DUSP4
IL6R
DUSP10
DUSP16
PEA15
DUSP1
MAP2K1
DUSP6
JAK1
DUSP9
DUSP5
DUSP2
MAPK3
MAPK1
MAP2K2
JAK2
DUSP7
PTPN11
TYK2
IL6ST
DUSP8
0
5000
10000
15000
List of genes
